# Effects of late-season sheep grazing following early-season steer grazing on population dynamics of sericea lespedeza in the Kansas Flint Hills

**DOI:** 10.1093/tas/txad037

**Published:** 2023-04-02

**Authors:** Jack E Lemmon, Walter H Fick, Jonathan A Alexander, Garth A Gatson, K C Olson

**Affiliations:** Department of Animal Sciences and Industry, Kansas State University, Manhattan, KS 66506, USA; Department of Agronomy, Kansas State University, Manhattan, KS 66506, USA; Department of Animal Sciences and Industry, Kansas State University, Manhattan, KS 66506, USA; Department of Animal Sciences and Industry, Kansas State University, Manhattan, KS 66506, USA; Department of Animal Sciences and Industry, Kansas State University, Manhattan, KS 66506, USA

**Keywords:** grazing, invasive species, *Lespedeza cuneata*, sheep, tallgrass prairie

## Abstract

Mature ewes were used in a 4-yr study to evaluate effects of intensive late-season sheep grazing on vigor of sericea lespedeza in native tallgrass prairie. Pastures (*N* = 8; 31 ± 3.6 ha) infested with sericea lespedeza (initial basal frequency = 1.4%) were assigned randomly to one of two treatments: early-season beef steer grazing (1.1 ha/steer; initial BW = 258 ± 34 kg) from April 15 to July 15 followed by no grazing for the rest of the year (control; **STR**) or steer grazing from April 15 to July 15 followed by intensive grazing by mature ewes (0.2 ha/ewe; **SHP**) from August 1 to October 1. Ewes (initial BW = 65 ± 3.1 kg) were assigned randomly to graze four of eight pastures; remaining pastures were not grazed from August 1 to October 1. Vegetation responses to treatment were measured along four permanent 100-m transects in each pasture. Herbivory on sericea lespedeza was monitored weekly in each pasture from July 21 to October 7. Herbivory on sericea lespedeza in SHP and STR after steer grazing and before sheep grazing was not different (*P* = 0.51). In contrast, sericea lespedeza herbivory following sheep grazing was greater (*P* < 0.01) in SHP than in STR. Herbivory of individual sericea plants was greater (*P* < 0.01) in SHP than in STR by the end of week 1 of the sheep-grazing period (10.6% vs. 0.5%); moreover, herbivory on sericea lespedeza steadily increased (*P* ≤ 0.01) such that 92.1% of sericea lespedeza plants were grazed in SHP compared to 1.4% in STR by week 8 of the sheep-grazing period. Whole-plant DM weight of sericea lespedeza at dormancy was less (*P* < 0.01) in SHP than in STR. Additionally, annual seed production by sericea lespedeza was less (*P* < 0.01) in SHP than in STR (114 vs. 864 seeds/plant). Pasture forage biomass was not different (*P* = 0.76) between SHP and STR after the steer-grazing period. Conversely, STR had more (*P* < 0.01) residual forage biomass than SHP at the end of the sheep-grazing period. Growth performance of beef steers grazing from April 15 to July 15 annually was not different (*P* ≥ 0.59) between treatments. Our results were interpreted to suggest that intensive late-season grazing by sheep decreased vigor of sericea lespedeza. Late-season sheep grazing decreased forage biomass by 904 kg DM/ha compared with late-season rest; however, residual biomass was adequate to prevent soil-moisture loss and erosion during the dormant season.

## INTRODUCTION

Sericea lespedeza (*Lespedeza cuneata*) is a high-tannin, invasive, perennial forb in the tallgrass prairie ecosystem (Eckerle et al., 2010). Sericea lespedeza is native to Southeast Asia, and was first introduced in the United States in 1896 as a potential forage crop. During the mid-20th century, it was used as a means of erosion control in Kansas (Ohlenbusch et al., 2007). Sericea lespedeza infestations reduce native grass production by up to 92% through a combination of aggressive growth, canopy dominance, prolific seed production, and allelopathy (Kalburtji and Mosjidis, 1992; Dudley and Fick, 2003; Eddy et al., 2003). In Kansas, sericea lespedeza infests approximately 250,000 ha of rangeland, primarily in the Flint Hills region (KDA, 2018). Infestations have negative impacts on native, ecologically important insects and grassland birds through habitat destruction and reduction of key feed resources (Silliman and Maccarone, 2005; Ogden et al., 2019).

If an invasive species is detected soon after initial establishment, eradication is possible; however, once it becomes environmentally adapted and is self-sustaining, eradication is usually not possible (Swanton and Booth, 2004). Herbicides are the most widely used means to control unwanted invasions of noxious weeds. Up to 25% of rangelands in the United States are treated with herbicides annually (Shaw, 1982; DiTomaso, 2000). Herbicides decelerate the spread of sericea lespedeza somewhat but application can be arduous, due to steep, rocky topography that is characteristic of the region. Decades of reliance on herbicides to combat sericea lespedeza have not resulted in satisfactory control (Wong et al., 2012). In addition, herbicides can be lethal to ecologically important, nontarget, native plant species and may cause further degradation of native habitat (Tunnell et al., 2006). This unintended loss in rangeland biodiversity can be difficult to reverse (Marshall, 2001).

Increased grazing pressure on sericea lespedeza by domestic herbivores may slow its spread and expedite some measure of biological control. Unfortunately, mature plants contain high levels of condensed tannins which reduce protein digestion by beef cattle and are a strong deterrent to grazing (Jones and Mangan, 1977; Eckerle et al. 2011a, 2011b, 2011c;Preedy et al., 2013). In contrast, small ruminants have greater tolerance for condensed tannins than beef cattle ( Robbins et al., 1991; Hart, 2001; Pacheco et al., 2012). Sheep, in particular, appear less susceptible to certain plant toxins than beef cattle and may be useful to selectively pressure noxious weeds like sericea lespedeza (Ralphs et al., 1991; Henderson et al., 2012).

Owensby et al. (2008) described the predominant grazing management practice in the Flint Hills region of Kansas as one that involves annual spring burning followed by intensive grazing with yearling beef cattle from April to August. During seasonal grazing, 40% to 60% of annual graminoid production is removed and pastures remain idle for the remainder of the year. During this time, the competitive, dominant, native plant species are temporally weakened through preferential grazing, thus creating a niche for invasive species to establish. If this cycle is repeated annually, invasive plants can severely degrade native ecosystems (Rambo and Faeth, 1999). Similarly, Eddy et al. (2003) reported that under this prevailing management practice, invasion by sericea lespedeza into the tallgrass prairie biome has steadily increased. Sericea lespedeza flowers and produces seed in late summer from August to September (Cope and Burns, 1974; Koger et al., 2002; Eckerle et al., 2010). The absence of grazing pressure during this interval strongly promotes seed production, seed distribution, and continued invasion of the Flint Hills ecoregion by this noxious weed. Wang et al. (2008) indicated that unchecked invasion by sericea lespedeza poses a serious threat to the cattle grazing industry; therefore, the objective of our experiment was to evaluate the effects of late-season sheep grazing following locally conventional steer grazing on vigor and reproductive capabilities of sericea lespedeza in the Tallgrass Prairie region.

## MATERIALS AND METHODS

The Kansas State University Institutional Animal Care and Use Committee reviewed and approved all animal handling and animal care practices used in our experiment. All animal procedures were conducted in accordance with the Guide for the Care and Use of Animals in Agricultural Research and Teaching (FASS, 2020).

### Location

The experiment was conducted during the growing seasons from 2013 to 2016 at the Kansas State University Bressner Range Research Unit located in Woodson County, Kansas. Native tallgrass pastures (*N* = 8; 31 ± 3.6 ha) infested with sericea lespedeza (initial basal frequency = 1.4%) were burned annually in early April. Pastures were assigned randomly to one of two treatments: early-season grazing with beef steers (1.1 ha/steer; initial BW = 258 ± 34 kg) from April 15 to July 15 followed by rest for the remainder of the year (control; **STR**) or steer grazing from April 15 to July 15 followed by intensive grazing with mature ewes (0.2 ha/ewe; **SHP**) from August 1 to October 1. Ewes (*N* = 808 ± 6; initial BW = 65 ± 3.1 kg) were assigned randomly to graze 4 of the 8 pastures; remaining pastures were not grazed from August 1 to October 1. Pasture treatment assignments were fixed for the 4-yr duration of the study.

### Animals

Yearling beef steers were obtained from various commercial cattle growers in southeastern Kansas from 2013 to 2016. Steers were weighed individually before grazing began each April and were assigned randomly to pastures to create a stocking density of approximately 1.1 ha/ steer. Steers were weighed individually again in late July after grazing was halted.

Mature ewes were obtained from two commercial sheep producers located in central and western Kansas. Ewes were transported to research site on approximately July 30 each year. Ewes were weighed immediately before grazing began on August 1 and immediately after grazing was halted on October 1. Final BW of sheep averaged 72 ± 3.1 kg. Sheep were monitored daily to assure they remained in assigned pastures and that fresh water was available continually. Death loss was <2% annually, and assumed to occur through predation or disease.

### Vegetation Responses

Vegetation responses to treatment were measured along 4 permanent 100-m transects in each pasture. Transects were laid out on a north–south gradient; ends were marked using steel t-posts. Immediately before and immediately after sheep grazing, a 100-m measuring tape was stretched from the southern end to the northern end of each transect. At 1-m intervals along each transect, above-ground plant biomass was measured using a visual obstruction technique (Robel et al., 1970).

Weekly estimates of herbivory were conducted to evaluate grazing pressure on select forb species in each pasture. The species of interest were sericea lespedeza, Baldwin’s ironweed (*Vernonia baldwinii*), and ragweed species (*Ambrosia artemisiifolia, A. bidentata,* and *A. psilostachya*). Individuals of each species or group of species (*N* = 100 per pasture weekly) were evaluated at temporary point transects. Point transect locations were determined randomly in control pastures. In treated pastures, point transects were located in areas where sheep grazing was observed to occur at the time of observation. Evidence of herbivory (i.e., obvious truncation of leaves or stems) on individual plants was recorded.

Plant species composition and soil cover were evaluated each October using a modified step-point technique (Owensby, 1973; Farney et al., 2017), along the permanent transects described previously. At 1-m intervals along each transect, 100 points were independently and randomly selected using a step-point device (Owensby, 1973). Each point was first categorized as a hit on bare soil, litter, or basal plant matter. Secondly, the closest rooted plant and the closest forb in a 180° arc in front of the selected point were recorded. These observations were then used to calculate the abundance of individual plant species via the method described by Farney et al. (2017). Pretreatment bare ground % (44% ± 1.3% for SHP and 47% ± 7.2% for STR), litter cover % (47% ± 2.6% for SHP and 46% ± 8.0% for STR), and basal plant cover % (8.7% ± 2.82% for SHP and 7.0% ± 1.29% for STR) were not different (*P* ≥ 0.63; data not shown) between treatments.

### Seed Production

A total of 100 mature sericea lespedeza plants were selected randomly and collected adjacent to permanent line transects in each pasture immediately after the first killing frost (approximately November 1 annually). Plants were placed into a labeled paper bag. Partial DM was measured using a forced-air oven (96 h; 55 °C). Individual plants in each sample were defoliated manually; seeds, chaff, and stems were placed into a South Dakota Seed Blower (E.L. Erickson Products, Model B; 10-cm tube) to separate seeds. Cleaned seed was weighed for each sample. Seed weight was converted to seed count assuming a density of 770 seeds/g (Vermeire et al., 2007; Vandevender, 2014). Average seed production was calculated by dividing the number of seeds by the number of sericea lespedeza plants in each sample (*N* = 100).

### Statistical Analyses

Line transect data were analyzed as a completely random design (PROC MIXED, SAS Inst. Inc., Cary, NC). Class variables included pasture, year, time (i.e., pretreatment or posttreatment), treatment, and transect. The model contained terms for treatment, time, and the two-way interaction. Year was considered a random effect. Weekly herbivory indices were also analyzed as a completely random design (PROC MIXED, SAS Inst. Inc., Cary, NC). Class variables included treatment, pasture, year, and week. The model contained terms for treatment, week, and the two-way interaction; year was considered a random effect.

Seed production by sericea lespedeza, DM weight of sericea lespedeza plants, soil cover, and plant species composition were analyzed as a completely random design, with treatment, pasture, and year as class variables (PROC MIXED, SAS Inst. Inc., Cary, NC). Models included a term for treatment only and year was considered a random effect.

Steer BW and ADG were analyzed as a completely random design (PROC MIXED, SAS Inst. Inc., Cary, NC). Class variables were treatment, pasture, and year. The model included a term for treatment only and year was considered a random effect. When protected by a significant F-test (*P* ≤ 0.05), means were separated using the method of Least Significant Difference. Least-squares means for the highest-order, significant (*P* ≤ 0.05) interaction term were reported.

## RESULTS AND DISCUSSION

**Figure 1.**
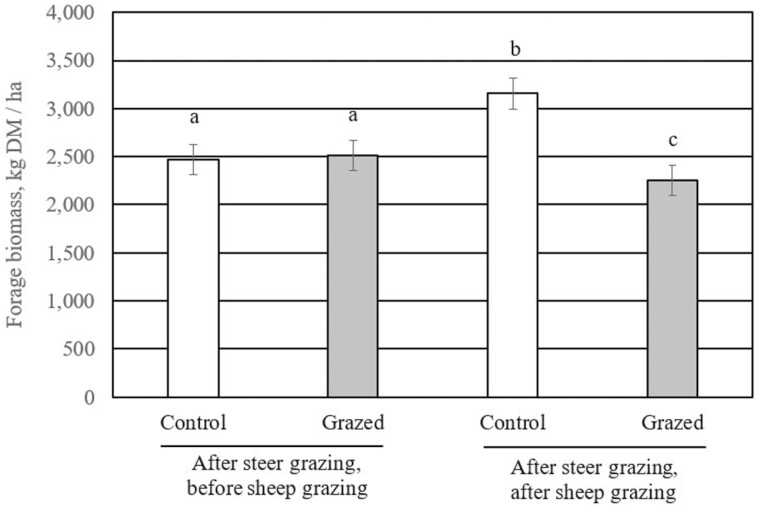
Effects of early-season grazing by beef steers followed by late-season grazing by sheep and time of measurement on pasture forage biomass [treatment × time − *P* < 0.01; ^a, b, c^ Columns with unlike superscripts differ (*P* < 0.05)]. Yearling steers were allowed to graze native tallgrass pastures (*N* = 4) from April 15 to July 15 from 2013 to 2016 (1.1 ha/ steer; initial BW = 258 ± 34 kg); pastures were not grazed for the remainder of the year (control). Yearling steers were allowed to graze native tallgrass pastures (*N* = 4) from April 15 to July 15 from 2013 to 2016 (1.1 ha/ steer; initial BW = 258 ± 34 kg); mature ewes grazed these pastures from August 1 to October 1 annually (0.2 ha/ewe; initial BW = 65 ± 3.1 kg; grazed).

Pasture forage biomass was not different (*P* = 0.29) between STR and SHP after steer grazing was halted and before sheep grazing began (Figure 1). Conversely, forage biomass on rested pastures was greater (*P* = 0.01) than that on SHP at the end of the sheep-grazing period. Similarly, Cassels et al. (1995) conducted a grazing study in the tallgrass prairie where different grazing systems and stocking rates were evaluated. They found that pastures that were grazed, followed by a period of rest had greater forage yield at the end of the growing season compared to those that did not.

Tannin content of sericea lespedeza peaks during August and September according to Eckerle et al. (2010) and Preedy et al. (2013). Condensed tannins are released during mastication and rapidly bind to plant proteins, rendering them unavailable to most herbivores during ruminal fermentation (Min et al., 2003). The likely aversion caused by this condition effectively protects the plant from herbivory by beef cattle prior to production and maturation of seed. In circumstances where beef cattle are the only significant source of herbivory, this allows sericea lespedeza to produce copious amounts of seed.

Herbivory of sericea lespedeza was not different (*P* = 0.99) and slight in STR and SHP immediately following the steer-grazing period (Table 1). In contrast, sericea lespedeza herbivory was greater (*P* ≤ 0.01) in SHP than in STR by the end of week 1 of the sheep-grazing period (10.6% vs. 0.5%); moreover, herbivory of sericea lespedeza steadily increased (*P* ≤ 0.01) over time such that 92.1% of sericea lespedeza plants were grazed in SHP compared to 1.4% in STR by week 8 of the sheep-grazing period. We interpreted these data to indicate that sheep displayed much greater preference for sericea lespedeza than steers. The lack of herbivory on sericea lespedeza prior to the onset of sheep grazing in our experiment was anticipated based on earlier reports. Sowers et al. (2019) indicated that sericea lespedeza was detectable only in trace amounts in yearling steer diets. Min et al. (2003) and Eckerle et al. (2011a) concluded that condensed tannins were a strong chemical deterrent to intake by beef cattle. Sowers et al. (2019) hypothesized that when yearling steers sampled small amounts of sericea lespedeza while grazing, the condensed tannins therein caused a flavor-related aversion causing them to avoid sericea lespedeza for the duration of the grazing season.

**Table 1.** Effect of late-season grazing by sheep on herbivory of sericea lespedeza cuneata (*Lespedeza cuneata;* treatment × week − *P* < 0.01, SE = 4.15)

Item	Steer grazing only^1^	Steer + sheep grazing^2^
Pre-Treatment^3^, % target species grazed	0.1^a^	0.6^a^
Week 1^4^, % target species grazed	0.5^a^	10.6^b^
Week 2^4^, % target species grazed	0.5^a^	22.4^c^
Week 3^4^, % target species grazed	0.9^a^	50.1^d^
Week 4^4^, % target species grazed	1.4^a^	64.8^e^
Week 5^4^, % target species grazed	2.5^a^	69.3^e^
Week 6^4^, % target species grazed	2.1^a^	78.4^f^
Week 7^4^, % target species grazed	3.5^a^	85.9^f,g^
Week 8^4^, % target species grazed	1.4^a^	92.1^g^

^1^Yearling steers were grazed on four pastures from April 15 to July 15 annually (1.1 ha/steer; initial BW = 258 ± 34 kg); pastures were not grazed for the remainder of the year.

^2^Yearling steers were grazed on four pastures (*N* = 8) from April 15 to July 15 annually (1.1 ha/steer; initial BW = 258 ± 34 kg); mature ewes grazed these pastures from August 1 to July 1 annually (0.2 ha/ewe; initial BW = 65 ± 3.1 kg).

^3^Percentage of sericea lespedeza plants showing evidence of defoliation immediately after yearling steers were removed and before sheep were allowed access to pastures.

^4^Percentage of sericea lespedeza plants showing evidence of defoliation each successive week during a 60-d period in which mature ewes were grazed on four pastures.

^a,b,c,d,e,f,g^Within row and column, means with unlike superscripts differ (*P* < 0.05).

Sheep also appeared to preferentially select other problematic forb species that steers avoided. Herbivory of Baldwin’s ironweed and ragweed spp. was not different (*P* ≥ 0.92) in STR and SHP immediately following the steer grazing period (Tables 2 and 3, respectively). Conversely, herbivory of individual Baldwin’s ironweed plants was greater (*P* ≤ 0.01) in SHP than in STR by the end of week 1 of the sheep-grazing period and was complete by the end of week 4. Sheep did not put a significant amount of grazing pressure on ragweed species until the end of week 2 of the sheep-grazing period; thereafter, herbivory of ragweed species steadily increased over time such that 57.6% of ragweed plants were grazed in SHP (*P* ≤ 0.01) compared to 0.6% in STR by the end of week 8 of the sheep-grazing period.

**Table 2.** Effect of late-season grazing by sheep on herbivory of Baldwin’s ironweed (*Vernonia baldwinii*; treatment × week − *P* < 0.01, SE = 3.69)

Item	Steer grazing only^1^	Steer + sheep grazing^2^
Pre-Treatment^3^, % target species grazed	9.9^a^	8.5^a^
Week 1^4^, % target species grazed	13.3^a^	64.8^d^
Week 2^4^, % target species grazed	21.4^b^	80.0^e^
Week 3^4^, % target species grazed	17.1^a,b,c^	92.8^f^
Week 4^4^, % target species grazed	19.1^a,b^	100^g^
Week 5^4^, % target species grazed	21.9^b,c^	99.8^g^
Week 6^4^, % target species grazed	17.1^a,b,c^	98.4^f,g^
Week 7^4^, % target species grazed	23.3^b,c^	100^g^
Week 8^4^, % target species grazed	25.8^c^	100^g^

^1^Yearling steers were grazed on four pastures from April 15 to July 15 annually (1.1 ha/steer; initial BW = 258 ± 34 kg); pastures were not grazed for the remainder of the year.

^2^Yearling steers were grazed on four pastures (*N* = 8) from April 15 to July 15 annually (1.1 ha/steer; initial BW = 258 ± 34 kg); mature ewes grazed these pastures from August 1 to July 1 annually (0.2 ha/ewe; initial BW = 65 ± 3.1 kg).

^3^Percentage of sericea lespedeza plants showing evidence of defoliation immediately after yearling steers were removed and before sheep were allowed access to pastures.

^4^Percentage of sericea lespedeza plants showing evidence of defoliation each successive week during a 60-d period in which mature ewes were grazed on four pastures.

^a,b,c,d,e,f,g^ Within row and column, means with unlike superscripts differ (*P* < 0.05).

**Table 3.** Effect of late-season grazing by sheep on herbivory of ragweed species (*Ambrosia psilostachya, A. bidentata,* and *A. artemisiifolia*; treatment × week − *P* < 0.01, SE = 4.35)

Item	Steer grazing only^1^	Steer + sheep grazing^2^
Pre-Treatment^3^, % target species grazed	0.1^a^	0.3^a^
Week 1^4^, % target species grazed	0.6^a^	5.8^a,b^
Week 2^4^, % target species grazed	0.2^a^	12.2^b^
Week 3^4^, % target species grazed	0.4^a^	23.4^c^
Week 4^4^, % target species grazed	0.7^a^	28.3^c^
Week 5^4^, % target species grazed	0.1^a^	41.0^d^
Week 6^4^, % target species grazed	2.0^a^	37.2^d^
Week 7^4^, % target species grazed	3.4^a^	55.2^e^
Week 8^4^, % target species grazed	0.6^a^	57.6^e^

^1^Yearling steers were grazed on four pastures from April 15 to July 15 annually (1.1 ha/steer; initial BW = 258 ± 34 kg); pastures were not grazed for the remainder of the year.

^2^Yearling steers were grazed on four pastures (*N* = 8) from April 15 to July 15 annually (1.1 ha/steer; initial BW = 258 ± 34 kg); mature ewes grazed these pastures from August 1 to July 1 annually (0.2 ha/ewe; initial BW = 65 ± 3.1 kg).

^3^Percentage of sericea lespedeza plants showing evidence of defoliation immediately after yearling steers were removed and before sheep were allowed access to pastures.

^4^Percentage of sericea lespedeza plants showing evidence of defoliation each successive week during a 60-d period in which mature ewes were grazed on four pastures.

^a,b,c,d,e,f,g^ Within row and column, means with unlike superscripts differ (*P* < 0.05).

Kremer (1993) reported that it is impossible to eradicate invasive plants without seed suppression. Suppression of seed production may be a key to achieving control of sericea lespedeza. Whole-plant weight immediately after the first killing frost averaged 3.1-fold less (*P* < 0.01) in SHP than STR over the duration of the study (Table 4). In addition, annual seed production by sericea lespedeza and total seed weight were less (*P* ≤ 0.01) in SHP than in STR. We concluded that late-season, intensive grazing by sheep may be an effective means for controlling the spread of sericea lespedeza.

**Table 4.** Effects of early-season grazing by beef steers followed by late-season grazing by sheep on whole-plant DM weight, total seed weight, and seed production of sericea lespedeza (*Lespedeza cuneata*), as measured immediately before seasonal plant dormancy

Item	Steer grazing only^1^	Steer + sheep grazing^2^	SE^3^	*P*-value^4^
Whole plant DM weight, mg/plant	4,424	1,443	357.6	< 0.01
Total seed weight, mg/ plant	1,123	148	85.6	< 0.01
Seeds, no./ plant	864	114	65.9	< 0.01

^1^Yearling steers grazed four pastures from April 15 to July 15 annually; pastures were rested for the remainder of the year.

^2^Yearling steers grazed four pastures from April 15 to July 15 annually; mature ewes grazed these pastures from August 1 to October 1 annually.

^3^Mixed-model standard error of the mean (SEM) associated with comparison of treatment main-effect means.

^4^Treatment main effect.

The success and proliferation of many invasive plants can be attributed to the volume of seed produced and to individual seed characteristics. Invasive plants tend to produce large amounts of small seeds that can remain nongerminated and viable for long periods in the seed-soil bank. Invasive-plant seeds tend also to germinate more readily than native-plant seeds (Ferreras and Galetto, 2010). Sericea lespedeza seed exhibits these characteristics. Its seed is very small, hard, and smooth. Established sericea lespedeza stands may produce 340 to 670 kg of seed/ha annually, with over 770,000 seeds/kg (Guernsey, 1970; Vermeire et al., 2007; Vandevender, 2014). Although sericea lespedeza was reported to have modest germination rates of 10% to 20% annually (Pieters, 1950), the number of seeds produced may overwhelm the seed-soil bank over time. Forty-year old sericea lespedeza seed recovered from the seed-soil bank was successfully germinated under laboratory conditions (Walters et al., 2005).

Grazing systems provide a management framework that allows plant communities to be influenced by the class and species of herbivore and the duration and frequency of grazing and rest. Hickman et al. (2004) stated that land managers are tasked with finding and implementing a grazing system that optimizes overall rangeland and animal productivity and health without compromising the integrity of the ecosystem. It is imperative that long-term rangeland health ramifications are considered. The grazing enterprise will not be sustainable if it damages or degrades the native ecosystem (White et al., 2000).

During our experiment, total basal vegetation cover increased (*P* < 0.01) in SHP pastures compared to STR pastures, (11.6% vs. 9.6% of total area; respectively), whereas litter cover on the surface of the soil and the amount of bare soil were not influenced (*P* ≥ 0.63) by treatment (Table 5). There was additional evidence that sheep grazing was associated with meaningful changes to the plant community at large. Basal area of all grasses and sedges was slightly greater (*P* = 0.01) in pastures grazed by cattle and sheep than in pastures grazed only by cattle (87.5% vs. 84.9% of total basal area, respectively). Conversely, major warm season grasses decreased in SHP pastures compared to STR pastures (36.9% vs. 43.9% of total C4 grass basal cover, respectively). Total basal cover of forbs was, likewise, less (*P* = 0.01) in SHP pastures grazed by cattle and sheep than in STR pastures grazed by cattle only, whereas basal cover of major wildflowers and shrubs was not different (*P* ≥ 0.20) between treatments.

**Table 5.** Effects of early-season grazing by beef steers followed by late-season grazing by sheep on occurrence of bare soil, litter cover, and basal plant cover in native tallgrass prairie infested with sericea lespedeza (*Lespedeza cuneata*)

Item	Steer grazing only^1^	Steer + sheep grazing^2^	SE^3^	*P*-value^4^
Bare soil, % of total area	29.9	29.5	3.30	0.91
Litter cover, % of total area	60.6	58.9	3.49	0.63
Basal vegetation cover, % of total area	9.6	11.6	0.75	< 0.01
Grass and sedge cover, % of total basal cover	84.9	87.5	1.04	0.01
C4 grasses^5^, % of total basal cover	43.9	36.9	2.29	< 0.01
Forb cover^6^, % of total basal cover	14.7	12.3	1.03	0.02
Major wildflowers^7^, % of total basal cover	1.06	0.75	0.236	0.20
Sericea lespedeza, % of total basal cover	3.62	2.12	0.633	0.02
Baldwin’s ironweed, % of total basal cover	0.82	0.57	0.149	0.09
Western ragweed, % of total basal cover	4.47	3.80	0.502	0.18
Shrub cover^8^, % of total basal cover	0.05	0.04	0.030	0.82

^1^Yearling steers grazed four pastures from April 15 to July 15 annually; pastures were rested for the remainder of the year.

^2^Yearling steers grazed four pastures from April 15 to July 15 annually; mature ewes grazed these pastures from 1 August to 1 October annually.

^3^Mixed-model standard error of the mean (SEM) associated with comparison of treatment main-effect means.

^4^Treatment main effect.

^5^Combined basal cover of big bluestem (*Andropogon gerardii*), little bluestem (*Schizachyium scoparium*), indiangrass (*Sorghastrum nutans*), and sideoats grama (*Bouteloua curtipendula*).

^6^Combined basal cover of all perennial and annual forbs.

^7^Combined basal cover of blue wildindigo (*Baptisia australis*), (catclaw sensitivebriar (*Mimosa nuttallii*), heath aster (*Symphyotrichum ericoides*), plains wildindigo (*B. bracteata*), prairie coneflower (*Ratibida columnifera*), purple poppymallow (*Callirhoe involucrate*), purple prairieclover (*Dalea purpurea*), and white prairie clover (*D. candida*).

^8^Combined basal cover of leadplant (Amorpha canescens), New Jersey tea (Ceanothus *americanus*), false indigobush (*Amorpha fruticosa*), smooth sumac (*Rus glabra*), buckbrush (*Symphoricarpos orbiculatus*), and prairie rose (*Rosa arkansana*).

Hickman et al. (2004) reported that the abundance of particular native plant species fluctuated from year-to-year as grazing intensity was manipulated. Sowers et al. (2019) found that yearling beef steers grazing in the tallgrass prairie consistently selected a diet of mostly graminoid species from May through July and only a minor component of forbs. In contrast, these researchers reported that the diet of mature ewes grazing tallgrass prairie contained greater proportions of forb species compared to beef cattle. The decline in total forb basal cover we observed may have been related to this divergent preference between herbivore types.

We reported that during the sheep grazing period, sheep showed strong selection preferences for sericea lespedeza, Baldwin’s ironweed, and native ragweed species (Tables 1, 2, and 3, respectively). In the case of sericea lespedeza, the selection translated to a meaningful decline (*P* = 0.02) in basal cover (Table 5). Alexander et al. (2021) and Duncan et al. (2021) indicated that repeated defoliation of sericea lespedeza over time resulted in decreased basal cover of the plant. Basal cover of Baldwin’s ironweed, in spite of heavy defoliation by sheep, only tended (*P* = 0.09) to be less in pastures grazed by sheep and cattle compared with pastures grazed only by cattle. In addition, basal cover of western ragweed was not influenced (*P* = 0.18) by treatment.

**Figure 2.**
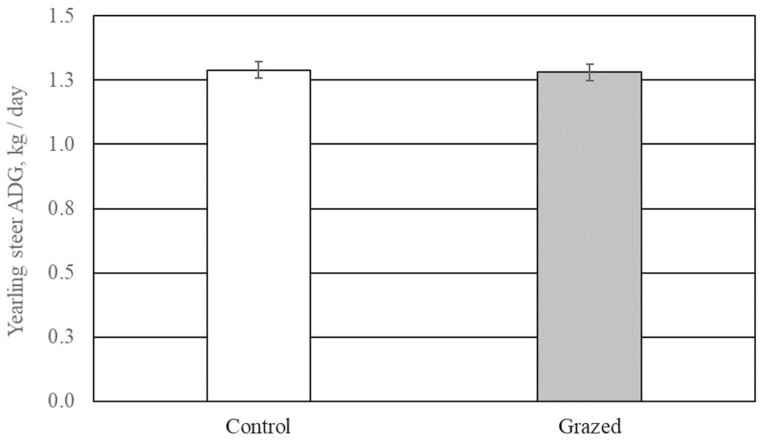
Effects of early-season grazing by beef steers followed by late-season grazing by sheep and time of measurement on yearling steer growth performance (treatment main effect - *P* = 0.73). Yearling steers were allowed to graze native tallgrass pastures (*N* = 4) from April 15 to July 15 from 2013 to 2016 (1.1 ha/ steer; initial BW = 258 ± 34 kg); pastures were not grazed for the remainder of the year (control). Yearling steers were allowed to graze native tallgrass pastures (*N* = 4 from April 15 to July 15 from 2013 to 2016 (1.1 ha/ steer; initial BW = 258 ± 34 kg); mature ewes grazed these pastures from August 1 to October 1 annually (0.2 ha/ewe; initial BW = 65 ± 3.1 kg; grazed).

Late-season, intensive sheep grazing on native tallgrass prairie appeared to decrease vigor and reproductive capabilities of sericea lespedeza. Sheep preferentially selected sericea lespedeza, Baldwin’s ironweed, and ragweed spp., whereas steers avoided these plants (Sowers et al., 2019). We interpreted herbivory patterns in pastures treated with late-season sheep grazing to indicate that condensed tannins in sericea lespedeza were not a deterrent to consumption by sheep. Late-season sheep grazing decreased forage biomass by 1,068 kg DM/ha compared with late-season rest; however, residual biomass on pastures grazed during the late growing season was likely sufficient to prevent soil-moisture loss, erosion during the dormant season, and application of prescribed fire the following spring. Although grazing pressure exerted by sheep was associated with noteworthy changes to the native plant community, it did not alter (*P* ≥ 0.59) growth performance of grazing yearling steers (Figure 2). The mature ewes used in our experiment gained a modest amount of body weight during the 60-d grazing bouts (initial BW = 65 ± 3.1 kg; final BW = 72 ± 3.1 kg). We concluded that late season grazing by sheep was consistent with responsible ecosystem stewardship and could be used to control sericea lespedeza and to add an additional, sustainable income stream to an existing ranching enterprise. Before implementing this management strategy, land managers should consider whether additional fencing and predator control may be needed.
